# “We're Still Here”: A Photovoice Study of Mothers' Perspectives 6 Years after the Flint Michigan Water Contamination Event

**DOI:** 10.1089/heq.2020.0135

**Published:** 2021-09-14

**Authors:** Melva Craft-Blacksheare, Marilyn S. Filter, Shan Parker

**Affiliations:** School of Nursing, University of Michigan-Flint, Flint, Michigan, USA.

**Keywords:** flint water crisis, photovoice, community-engaged research, maternal/child health

## Abstract

**Purpose:** This study explored the lived experiences of pregnant and parenting women after the water contamination event in Flint, Michigan (Flint Water Contamination Event [FWCE]), by helping them visualize their everyday challenges, assets, and family health concerns.

**Methods:** The photovoice methodology was utilized in this study. Individual participants met with researchers to discuss their photos using the SHOWeD method. Researchers analyzed the photographs, accompanying narratives, and discussion session transcripts using descriptive coding and thematic analysis.

**Results:** Data analysis revealed five themes: (1) having a healthy pregnancy and well children is a central concern in everyday life, (2) children serve as social and practical resources for family, (3) meeting children's need for safe water is a significant concern, (4) the community and its households battle to get safe affordable water, and (5) there are resources available in the community to support mothers.

**Conclusion:** While much information is still being collected and published in the aftermath of the FWCE, this study was the first to use the photovoice method to allow pregnant and parenting women to express their concerns about how the FWCE continues to affect the health and care of their families.

## Introduction

From the 1960s to 2014, the Detroit Water and Sewerage Department (DSWD) supplied treated and filtered water from Lake Huron to the city of Flint, Michigan. After terminating its contract with the DSWD, the city began to use water from the Flint River on April 25, 2014. After the switch, residents reported water discoloration and odor and endured three boil-water advisories due to elevated *Escherichia coli* and total coliform violations.^1^ Additionally, the city's failure to use anticorrosive material in the water, a practice mandated by the 1978 Clean Water Act, was linked to a Legionnaires' disease outbreak.^2^

Although Flint households reconnected to the DWSD pipeline in 2016, residents remained concerned about their exposure to toxic chemicals during the crisis. Their concerns were warranted. After reviewing Flint statistical data from 2008 to 2015, Grossman and Slusky determined that after April 2014, women's fertility rates decreased by 12% and fetal death rates increased by 58%.^3^ Furthermore, on average, babies were born 1 to 2 weeks earlier and weighed 150 g less during that period.^3^

The city is especially vulnerable to the effects of the Flint Water Contamination Event (FWCE) as the social determinants of health already contribute to poor health and health outcomes in this area.

As a 20th century U.S. Auto Town, Flint had one of the highest per capita incomes. Due to the closure of manufacturing plants (1980–1990), Flint experienced population losses, aggregate income decline, and increased unemployment rates of 16.9% in July 2009. Postrecession unemployment between 2011 and 2012 gradually declined between 8.3% and 11% through 2013. Presently, more than 40% of Flint's residents live below the poverty line.^4–6^

Furthermore, neighborhood challenges persist in Flint following decades of public and private city disinvestment, high unemployment and poverty rates, struggling public schools, significant street violence, and a history of racial segregation. Despite resources and continued efforts (neighborhood initiatives and institutional support) to improve the residents' plight, effects of the FWCE persist, even 6 years later. These dangers are supported by evidence showing that high-risk populations, including pregnant women and developing fetuses and with low socioeconomic status, are more likely to be exposed to pollutants (such as contaminated water) associated with preterm birth, low birth weight, and stillbirth.^7^

Given these concerns, the aims of this study were to use a photovoice methodology and qualitative interviews to explore pregnant and parenting mothers' experiences of their environment related to their families' health after the FCWE, their everyday challenges and assets, and how these perspectives reflect the FWCE legacy. Furthermore, this study focused on how the FWCE continues to affect women's lives, focusing on the physical and social structures that have either improved or worsened their families' overall health.

The photovoice method creates opportunities for participants to record and reflect on the challenges and assets they encounter in their everyday community experiences. Photovoice is widely employed in public health and nursing research in a variety of global settings.^8–14^ This method also has been used to address such public health issues as maternal and child health, community disaster legacies, youth violence, and Flint community building.^9,11,15,16^ Finally, photovoice draws upon a documentary photography tradition, which includes an array of styles, genres, and purposes.

## Methods

### Study design

Data were collected through in-depth interviews and photos gathered using the photovoice method.^8^ The aim was to highlight the lived experience of pregnant and parenting mothers since the FWCE.

#### Ethical considerations

The University of Michigan-Flint Institutional Review Board approved all study procedures. All participants attended a study presentation before signing a consent form for participation. All participant pictures are used with permission.

#### Sample, setting, and recruitment

Inclusion criteria were English speakers, at least 18 years of age, who were pregnant or parenting in the previous 3 years and living in Flint during the FWCE. Initially, researchers contacted 30 women who expressed interest in the study, 29 of whom were found eligible for participation. Nine participants, selected through purposeful sampling, completed all study requirements. Purposive sampling allowed researchers to decide what needs to be known and set out to find people who can and are willing to participate and provide information by virtue or knowledge.^17^

The two clinics used as recruitment sites were part of a federally qualified health center (FQHC). Through multiple clinic sites located in the zip codes most affected by the FWCE, these clinics provide care for more than 97,000 Flint residents.

Face-to-face recruitment was conducted by researchers and two graduate student research assistants trained in human subjects' research methods and study-specific tasks. At clinic site one, certified nurse midwives explained the study to women attending prenatal classes and then followed up with those interested through phone. At clinic site two, the research team recruited women from the prenatal and pediatric clinics' waiting areas. After completing interest forms, potential participants were called by the study team to schedule their attendance at informational sessions.

#### Procedures

Potential participants were invited to an informational group session, during which researchers presented the study purpose and intent and discussed ethical considerations in photovoice methods, covering issues of privacy, safety, autonomy, and representation, before asking them to review and sign the study consent form. Each participant received a copy of their signed consent form in addition to consent forms for potential photographed individuals. A professional photographer shared tips for taking meaningful pictures. Participants received digital cameras and practiced taking pictures. The photographer analyzed the photos and suggested tips to improve quality. All pictures were taken with the provided digital cameras; personal cell phones were not used. Researchers asked participants to photograph everyday life situations that showed their personal challenges and successes as pregnant women and parenting mothers during and after the FWCE.

At the post data collection session 2 weeks later, participants met individually with a researcher to review and discuss their photographs. Researchers analyzed collected data using techniques suggested for photovoice analysis by Wang and Burris.^8^ Each researcher took extensive notes using the *SHOWeD* framework, which consists of five questions: (1) What do you see here? (2) What is happening here? (3) How does this relate to our lives/your life? (4) Why does this problem or strength exist? (5) What can we do with or about it?^8,10^

Participants chose the most meaningful photographs and developed the stories they wanted to present. Researchers reviewed the narrative with the participants for accuracy and detail, writing extensive notes to reflect their resources, strengths, and stories. After returning the cameras, participants received a $50.00 gift certificate to show appreciation for their contributions.

#### Data analysis

The researchers used descriptive coding and thematic analysis (Braun and Clarke) in a systematic manner across the entire data set to convert collected data into initial codes and themes.^18^ The four-member research team met on five occasions to review photographs, narratives, and field notes. Data configuration began by familiarizing the members of the research team with the data through multiple readings, discussion, and generation of codes. Continuous rereading and group discussion of participant's verbatim dialog, review of field notes, and active discussion forged agreement of codes among researchers. Accepted codes were then collated into potential themes, gathering all relative data until saturation was noted. All researchers reviewed and approved the coding and thematic analysis. Data triangulation was achieved using the photos, participant interviews, and field notes to determine a comprehensive understanding of the results, thus allowing the researcher to capture a more complete portrait of the phenomena of parenting during the FWCE.

## Results

The majority of participants identified as African American, with one identifying as non-Hispanic white. Most participants resided in the two Flint city zip codes that have the highest poverty rates and residents with the lowest social economic status.^4(p.145)^ The findings included five key subthemes under the overarching theme of children, family, community, and resources.

### Theme 1: Having a healthy pregnancy and well children is a central concern in everyday life

**FIG. 1.**
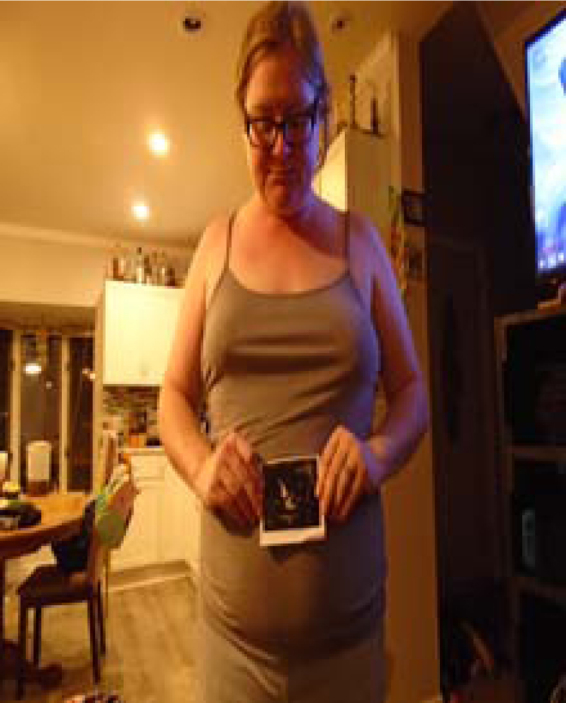
Visual Confirmation.

**FIG. 2.**
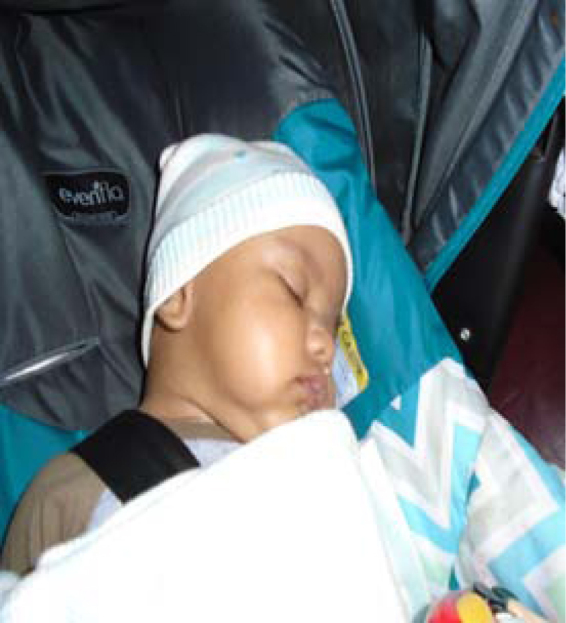
An Easy Baby.

Participants clearly identified their children's well-being as the main source of positivity and optimism in their lives. Furthermore, the women valued motherhood and viewed their children as the basis for their happiness and strength, the strongest reason for persevering in difficult situations, and their main source of hope for the future. Figure 1, titled *Visual Confirmation*, shows a participant with an ultrasound picture of her baby, which served as evidence that her pregnancy was progressing well. Another mother described her 2-month-old infant as “easy,” with a photo (Fig. 2, *An Easy Baby)* of her infant sleeping peacefully. She also said she wanted more children because “every kid makes life better,” but cannot afford another due to the FWCE.

### Theme 2: Children serve as social and practical resources for family life

**FIG. 3.**
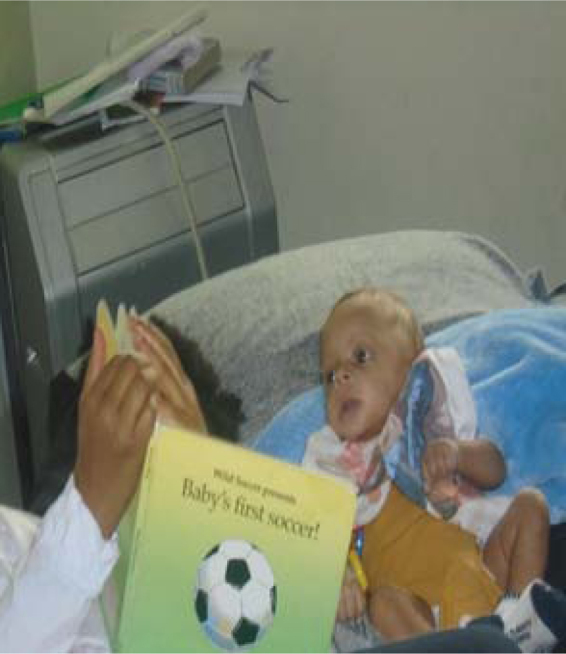
Big Sister Reads to the Baby.

In particular, mothers cherished their children and remarked that their older children took care of and bonded with their younger siblings, recognized their roles in solidifying family bonds, contributed to their younger siblings' development, and helped take pressure off their mothers. In Figure 3, *Big Sister Reads to the Baby*, the mother recognized the bond between her children, a relationship she encouraged. After expressing hope that the family would continue to help each other, this mother stated,

I didn't read to them [older children], but it amazes me how [my daughter] would see me reading to her baby brother, and… she just picked it up and began to do it, too. God, family, and education are important in our lives. I didn't know education was a strength at first, until I saw my daughter doing it… nothing is more important than family.

### Theme 3: Meeting children's need for safe water is a significant concern

**FIG. 4.**
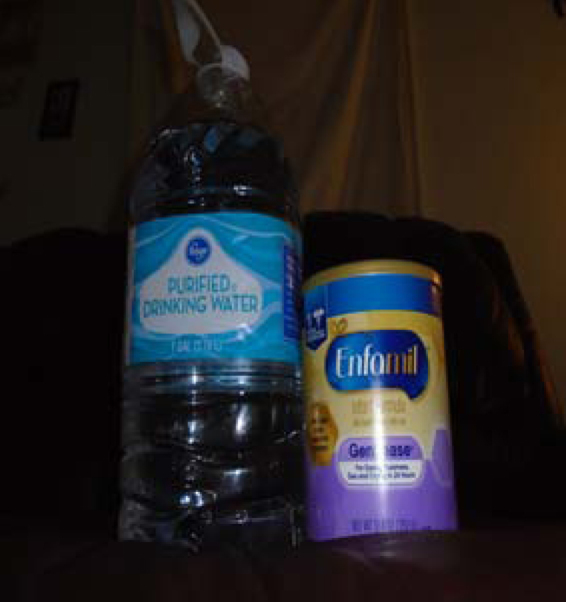
The Process to Get my Baby to Eat.

Participants were concerned about having safe water for their children and mentioned the health effects of consuming contaminated water. Figure 4, *The Process to Get my Baby to Eat*, illustrates the challenges this mother experienced trying to feed her baby safely. While making safe formula amid the FWCE continues to be a challenge, this time-consuming multistep process is necessary to ensure her baby's health. The woman stated,

I buy nine or 10 cans of formula and the water about once a week just for the baby. I have to buy water to make formula. If I had good tap water, I could just make it at home. I get the formula at the corner store and the water at Kroger… When they fix the pipes, is the water good to drink or not? They should give bottled water away again until everything is fixed and safe, and we shouldn't have to keep paying for water we can't use.

**FIG. 5.**
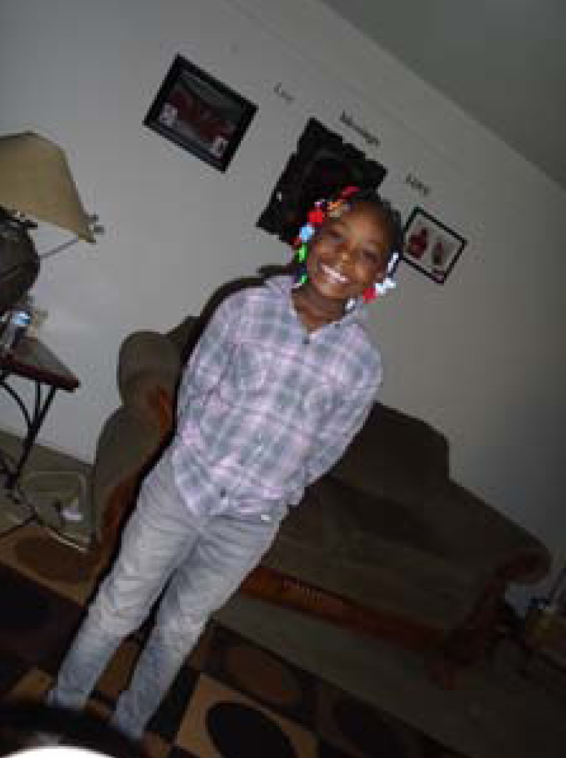
Water and Skin.

Figure 5, *Water and Skin*, highlights one mother's concern about skin irritation related to the FCWE. This mother discussed the time and effort involved in ensuring her daughter has healthy skin, including using special soaps and lotions and trying to see a dermatologist. Relating skin issues to poor water quality, this mother stated,

She's happy… [but] she has been complaining about her legs and has a whole bunch of bumps, dry skin, and burning in the bath. We have changed soaps and laundry detergents. I try to keep it moist and use a special kind of lotion.

### Theme 4: The community and its households battle to get safe affordable water

**FIG. 6.**
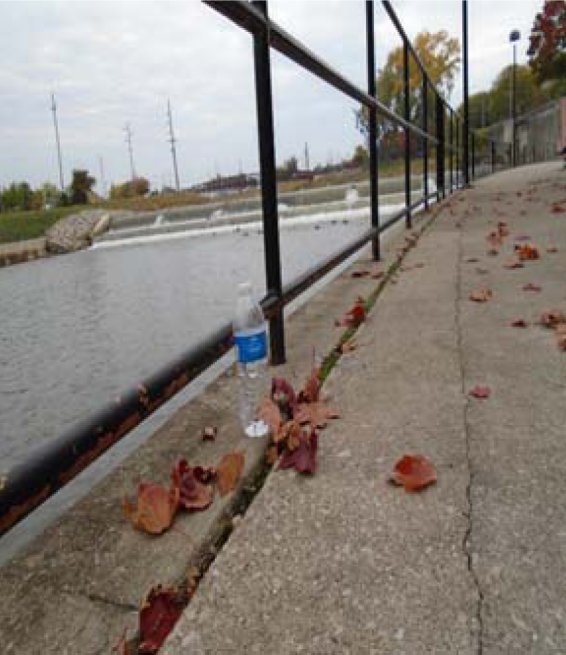
The Battle for Clean Water.

Participants described the struggle to get safe affordable water, often linking such water to their children's well-being. The participant who took the picture for Figure 6, *The Battle for Clean Water*, stated,

This is a picture of a water bottle in front of the Flint River. There is free water in the background, but we can't use it. We have to drink/use bottled water. We need water… we can't survive without it. But with the Flint water, we can't wash up. Without clean water, what can we do?

**FIG. 7.**
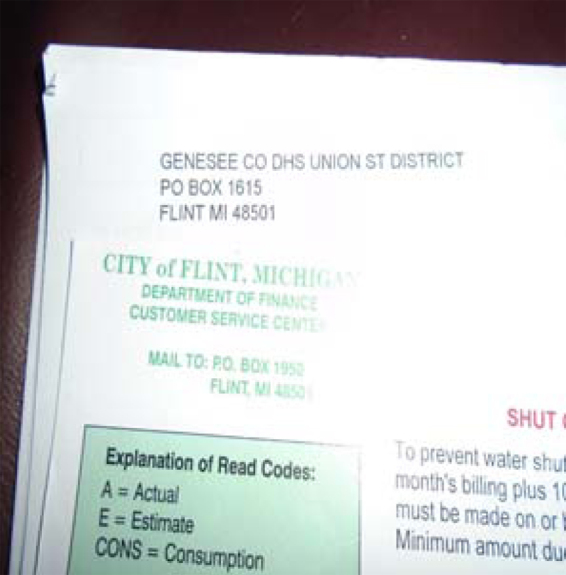
Water Bills and Shutoffs.

Figure 7, *Water Bills and Shutoffs*, shows a shutoff notice some Flint residents received during the FWCE. Residents were upset that they were charged for something that was unsafe and unhealthy. This participant stated,

This is our third shutoff in a row. I paid… [but] I'm not going to keep paying for water I can't use. It's about $250 a month. We shouldn't be charged for water until there is a real fix and the water is safe. Maybe DHHS can help. Maybe if we could have a strike against water bills, like the workers at GM…I don't think anyone is organizing now, but they did a few years ago, somewhat.

### Theme 5: There are resources available in the community to support mothers

**FIG. 8.**
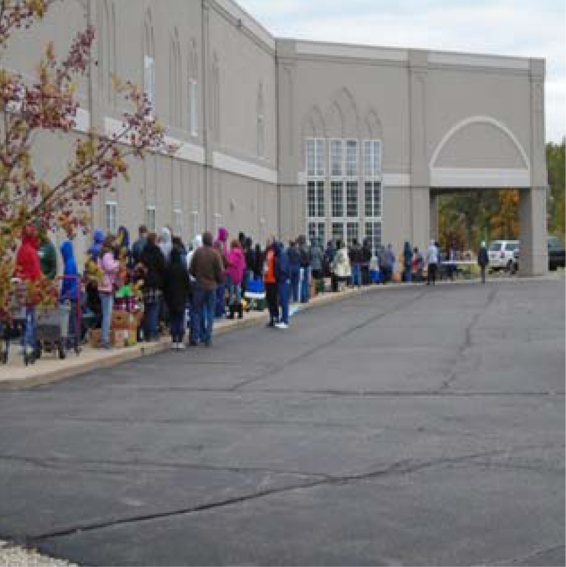
A Food Giveaway.

Participants described receiving support from their children, other family members, strangers, churches, public agencies, institutions, and natural features. Figure 8, *A Food Giveaway*, shows a common scene in Flint—people in line for food distribution. This participant stated,

This is a picture of a food giveaway at a church in Flint. Places like this serve as a resource for me and my family. We are able to get food and things that we need to help us stretch our money or to support us when we don't have enough money for the month. I often see some of the same people [here]… we support each other and share information about other resources in the community. There are a lot of people in these lines. People need help! They need these resources in order to live.

## Discussion

Through their photos and narratives, these study participants expressed their everyday challenges and assets related to the legacy of the FWCE. Recurrent issues that emerged were valuing motherhood, cherishing children and family members, persistent water fears, distrust of government, and neighbors helping neighbors.

### Valuing motherhood

The participants reported that their children were the most valued and meaningful part of their lives, as well as their top daily priority. Furthermore, their children provided a framework for their life purpose, everyday tasks, roles, responsibilities, strengths, and abilities. With these participants placing such value on children and mothering, these findings show how low-income women—who may have few opportunities to be valued and socially recognized—are able to establish a positive identity. In the absence of other available projects (e.g., education, work, and community leadership or participation), caring for their children filled their days. In fact, these mothers did not consider waiting until they met middle-class standards of security to have children. One participant said, “Waiting for the right time risks the right time not coming.” Similarly, Edin and Kefalas reported that many low-income mothers see children as the best part of life, often crediting their children for virtually every positive aspect of their lives. For these women, motherhood truly was a way to construct positive meaning in otherwise stressful lives. Moreover, they reported valuing motherhood more than marriage, schooling, and employment.^19^

Dodson and others argued that introduction of strict work requirements for social assistance in the mid-1990s revealed women's moral economy of valuing their children's emotional security and safety over the welfare bureaucracy's demands that they need to find employment.^20^ Moreover, children's centrality to poor mothers' identity formation and everyday routines has been obscured by the stigma against single motherhood created in public discourse and these mothers' perceived need to opt out of social research participation to avoid the potential material problems doing so could create.^21^

### Cherishing children and family members

Participants reported that their children served as practical resources for their families. In addition, participants reported that their families were of utmost importance to them. Family included not just blood relatives but also other loved ones, including significant others, parents, siblings, and cousins. These participants wanted all of their loved ones to have high-quality water and health care. While there were limited mentions of receiving financial and caregiving support from family members, the women reported receiving nonmonetary support from and sharing information with nonrelative “community kin” in other neighborhood settings.

### Persistent water fears

The women in this study discussed the unknown, long-term health outcomes from exposure to contaminated water. According to the Toxic Substances and Disease Registry, ∼99% of lead taken into an adult's body is excreted within a few weeks, compared with only about 32% of lead taken into a child's body. Indeed, there are no safe levels of this powerful neurotoxin.^22^ The women also discussed the lack of drinking water in schools, showing photos of drinking fountains with signs indicating there was no water. Moreover, participants mentioned the dearth of bottled water and their fear of bathing children in brown water, which stains sinks and tubs.

### Distrust of government

Another common topic was distrust of the three governmental systems involved in the FWCE: the city of Flint, the state of Michigan, and the U.S. federal government. The women felt they were given limited, inconsistent, and sometimes false information about the effects of Flint River water. Government officials told participants that the water was safe for drinking, cooking, and bathing. Additionally, participants mentioned how the state-supported children's health insurance and utility bill assistance programs require enrollment forms to be completed using a computer. Unfortunately, many of these women did not have computer access and were unable to enroll with smartphones that did support the computer application. This issue highlights the disconnect between governmental agencies and the people they serve. The theme of distrust of governmental agencies was further supported by water cost increases, which made it prohibitive for many participants to pay their water bills. Finally, distrust of the government extended to health care. For example, one participant, concerned about skin rashes her daughter experienced after bathing, was denied a dermatologist referral.

### Neighbors helping neighbors

Finally, women described how neighbors helped each other, such as giving rides to the water distribution center. In one neighborhood, a person bought and adjusted a semi-box to collect and dispense rainwater. Participants also bonded during study participation by interacting during the informational session and giving each other rides to the interview session. Clearly, these actions indicated the women's willingness to rely on each other to meet their daily needs.

### Limitations

Several limitations should be noted in interpreting this study. The women were not asked directly whether they received support from their partners, the children's father, or extended family. Instead, the researchers focused on each woman's personal stories, giving attention and weight to their individual perceptions. Another study limitation was the small sample size. While 29 eligible women were interested in the study, only nine participants completed it. To address the common problem of lack of transportation to the study site, the researchers offered participants bus passes, taxi rides, and personal rides. Finally, frequent changes in cell phone contact information (due to disconnected cell phones) made it impossible to maintain contact with some individuals.

## Conclusion

At its core, photovoice is a form of Participatory Action Research concerned with democratizing and using popular knowledge as a basis for social justice action. Through the SHOWeD photo interpretation method, participants told their stories in detail, illustrating the FWCE's lasting effects on their lives. These women described their love for their children; the joy, struggles, and concerns of daily living; and the often-suppressed fears about their families' health. Ultimately, these women want real answers and support from their government officials so they can feel that this situation will end and allow them to feel safe again. Future recommendations for the project include a public display of photos to share with the Flint and university community. The researchers would consider a follow-up study of women in the Flint community who were parenting during the Covid-19 pandemic.

## Abbreviations Used

DSWD: Detroit Water and Sewerage Department
FWCE: Flint Water Contamination Event
